# Applications of research evidence during processes to acquire approvals for syringe services program implementation in rural counties in Kentucky

**DOI:** 10.1080/07853890.2022.2028001

**Published:** 2022-01-31

**Authors:** Sean T. Allen, Suzanne M. Grieb, Jennifer L. Glick, Rebecca Hamilton White, Tyler Puryear, Katherine C. Smith, Brian W. Weir, Susan G. Sherman

**Affiliations:** a Department of Health, Behavior and Society at the Johns Hopkins Bloomberg School of Public Health, Baltimore, MD, USA; b Center for Child and Community Health Research, Department of Pediatrics, Johns Hopkins School of Medicine, Baltimore, MD, USA

**Keywords:** Injection drug use, syringe services programs, research evidence, rural, drug policy

## Abstract

**Introduction:**

Despite decades of empirical research in the US and internationally documenting the benefits of implementing syringe services programs (SSPs), their implementation may be controversial in many jurisdictions. Better understanding how research evidence is applied during SSP implementation processes may enable the public health workforce to advocate for program scale up. This study explores applications of research evidence during processes to acquire approvals for SSP implementation in rural counties in Kentucky.

**Methods:**

In-depth interviews were conducted among eighteen stakeholders (e.g. health department directors, SSP operators) involved in SSP implementation in rural Kentucky counties. Stakeholders were asked to describe the contexts surrounding SSP implementation processes. Interviews were transcribed and analysed for applications of research evidence. Research evidence-related quotes were subsequently categorised based on the typologies for applications of research evidence developed by Weiss *et al.* (instrumental, conceptual, and symbolic) and a fourth category for instances when research evidence was not used.

**Results:**

Instrumental applications of research evidence occurred at the intrapersonal and interpersonal levels to dispel concerns about SSPs and formed the basis for implementation support. SSP proponents used research evidence in a conceptual manner to address underlying attitudes and beliefs that were not evidence-based. Participants reported symbolic research evidence applications to justify pre-existing attitudes and beliefs about meeting the public health needs of people who inject drugs. Lastly, in some instances, research evidence was met with scepticism and an unwillingness to consider its merits.

**Conclusion:**

Applications of research evidence during SSP implementation approval processes in rural Kentucky counties were heterogeneous in nature. Better understanding the diversity of ways in which research evidence may be employed during SSP implementation processes may support efforts to improve the public health of people who inject drugs.

**Key messages**
1. Applications of research evidence during SSP implementation approval processes in rural Kentucky counties were heterogeneous in nature.
2. Instrumental applications of research evidence occurred at the intrapersonal and interpersonal levels to dispel concerns about SSPs and formed the basis for implementation support.
3. SSP proponents used research evidence in a conceptual manner to address underlying attitudes and beliefs that were not evidence-based.

## Introduction

Throughout the world, people who inject drugs (PWID) have been disproportionately affected by blood-borne infections, including hepatitis C virus (HCV) and human immunodeficiency virus (HIV) [1–4]. A recent analysis estimated that 17.8% of PWID worldwide are living with HIV and more than half are HCV-antibody positive [2]. Sharing injection equipment (e.g. syringes, cookers) is a primary route of infectious disease transmission among PWID [5,6]. While there are many interventions aimed at preventing HIV/HCV transmission, syringe services programs (SSPs), sometimes referred to as harm reduction programs or needle and syringe exchange programs, remain one of the most effective strategies to prevent new infections among PWID [7–11]. Several decades of global research have demonstrated that SSPs are not only cost-effective, but also effective conduits to link PWID to drug treatment and other health services [7,12–15].

In the United States (US), SSPs were first implemented in the 1980s; however, their implementation has not been without controversy [16]. For example, SSP implementation may be obstructed by opposition from policymakers, law enforcement, and community members [17–19]. There are a number of interrelated and overlapping drivers of SSP opposition, including: fear, stigma, misunderstanding, and “not in my back yard” attitudes and beliefs about the provision of substance use-related services. SSP opposition has led to many policy-level challenges for program implementation [17–25]. For example, SSP operations in the District of Columbia were once subject to a buffer zone policy that prohibited operations within 1,000 feet of a school [19,20]. In other jurisdictions, SSP operations may be obstructed by drug paraphernalia laws, funding restrictions, and policies that prevent SSPs from following evidence-based best practices [17,18,24,26]. Given recent escalations in injection drug use-associated morbidity and mortality, there is an on-going need for communities to remove policy-level barriers to SSP implementation and ensure PWID have access to the services they require.

Better understanding how research evidence is used or not used during policy change processes to support SSP implementation may afford the public health workforce enhanced capacity to advocate for programs that protect communities from injection drug use-associated morbidity and mortality. There are many frameworks that examine the mechanisms underlying policy change processes; Weiss *et al.*, for example, developed typologies for understanding applications of research evaluation that have been widely cited in the health policy and evaluation literature [27–32]. As succinctly stated by Weiss *et al.*, “Evaluations could be used (a) instrumentally, to give direction to policy and practice; (b) politically or symbolically, to justify pre-existing preferences and actions, and (c) conceptually, to provide new generalisations, ideas, or concepts that are useful for making sense of the policy scene” [27]. It should also be noted that research evidence is sometimes not used in policymaking processes or may be used in ways that are context-dependent [26,27]. A 2015 study that examined the role of research evidence in shaping policy change processes for SSP implementation in three urban-based US cities, for instance, found that there was a range of ways in which stakeholders (e.g. policymakers, advocates for SSP implementation) applied research, and that, in some instances, research was met by policymakers who were unwilling to incorporate it into their policy discussions [26].

Many studies have examined the relationship between SSP implementation and the public health of PWID populations in urban areas, but few have been conducted in rural areas. This reflects a significant gap in the literature given that metropolitan-based research may not be directly translatable to rural communities responding to the modern opioid overdose crisis. In addition, there is considerable heterogeneity in both the degree to which rural communities have access to evidence-based response strategies (e.g. medications for opioid use disorder, SSPs) and the magnitude with which they have been affected by injection drug use-associated morbidity and mortality [33–36]. For instance, rates of opioid-related inpatient hospital stays vary considerably between rural states (e.g. in 2014, Kentucky and Iowa had rates of 280.4 and 72.7 per 100,000 opioid-related inpatient hospital stays, respectively) [37]. Exploring SSP implementation processes in rural communities represents an important realm of scientific inquiry, particularly because research has found that risks for infectious disease outbreaks among PWID are concentrated in non-urban counties [38].

Following the 2015 HIV/HCV outbreak among PWID in rural Scott County, Indiana (US), several predominantly rural states experienced rapid proliferations of SSP implementation. In 2015, legislation was passed in Kentucky to allow communities to implement SSPs [39]. However, the legislation required three approvals prior to program implementation: the Board of Health at a local health department, county fiscal courts, and city councils. Despite requiring multiple levels of approval to implement SSPs, in 2021, more than 70 SSPs were operational across Kentucky with many located in rural counties identified as vulnerable to injection drug use-associated infectious disease outbreaks [39]. The legislative requirement to obtain three levels of approval for SSP implementation paired with the proliferation of programs across rural Kentucky presents a unique opportunity to explore how stakeholders (e.g. advocates for SSP implementation, health department directors) employed research evidence during processes to secure approvals for SSP operations. The purpose of this research is to explore applications of research evidence during processes to acquire approvals for SSP implementation in rural counties in Kentucky.

## Methods

### Data collection

From August-October 2020, in-depth, semi-structured interviews were conducted with 18 persons involved in the implementation of SSPs in rural counties throughout Kentucky. Participants had varying degrees of involvement with SSP implementation (e.g. health department directors who advocated for SSP implementation and later launched programs, healthcare providers who advocated for program implementation, and SSP operators). To identify potential participants, we conducted comprehensive searches of publicly available literature (e.g. media reports, governmental reports) related to SSPs in Kentucky. Potential participants were also identified over the course of interviews *via* participants describing others who played a role in SSP implementation. Persons identified during interviews were then vetted against public records to confirm their potential role in SSP implementation. Eligibility criteria included having played a role in SSP implementation and being at least 18 years of age.

Stakeholders were contacted *via* e-mail, informed about the study, and asked if they would be willing to participate. For persons who expressed interest, interviews were scheduled *via* Zoom or phone. All interviews were conducted by the first author, who is from southeastern Kentucky, familiar with issues related to SSP implementation in rural areas, and has conducted several previous studies related to SSPs, harm reduction, drug policy, and rural health disparities. Prior to beginning the interview, potential participants were able to learn more about the purpose of our study and ask questions. Given the potential sensitive nature of our interviews, we elected for an oral consent process; all participants provided oral consent before interviews were initiated. Each interview lasted approximately 45 min and was audio recorded. Prior to beginning the interviews, we informed potential participants that we would offer them a $25 gift card as an incentive for their participation. Interviews and initial analysis were occurring simultaneously, and recruitment stopped when content saturation was achieved (i.e. the Principal Investigator heard similar responses from participants and interviews did not yield new insights or afford nuanced understandings) [40]. The Institutional Review Board at the Johns Hopkins Bloomberg School of Public Health approved this study.

### Interview guide

Broadly, the parent study of which this analysis is a part aimed to understand the barriers and facilitators to SSP implementation in rural counties in Kentucky. The interview guide for the parent study was informed by both the Consolidated Framework for Implementation Research (CFIR) and Kingdon’s multiple streams model of policy change given the potential complexities associated with SSP implementation in Kentucky (e.g. acquiring three levels of approval, addressing underlying concerns about SSPs, and changing policies to support SSP operations) [41,42]. The CFIR was well-suited for the parent study purpose because it provides a systematic way to explore potential barriers and facilitators for the implementation of an innovation [41]. The CFIR consists of five domains (intervention characteristics, outer setting, inner setting, characteristics of individuals, and process) and each has multiple constructs [41]. We asked participants to broadly describe the process of SSP implementation in their communities and subsequently asked follow-up questions to ascertain nuance that reflected relevant constructs of the CFIR. In addition, given that SSP implementation often requires policy changes (e.g. decriminalising drug paraphernalia), we also used Kingdon’s multiple streams model of policy change to inform our interview guide for the parent study [42]. This model suggests that policy changes occur when three streams align: a problem stream, a policy stream, and a politics stream [42]. In instances where participants mentioned policy change, we asked probing questions to glean in-depth understandings of how policy changes were achieved. After developing a preliminary interview guide, we piloted the instrument internally with our study team and refined items as needed. In addition, participants were asked to describe their roles during SSP implementation (e.g. program implementer, health department director).

### Analysis

Audio recordings were transcribed verbatim, and transcripts were cleaned of any identifying information (e.g. names or references to specific places). An initial coding scheme was developed using a list of *a priori* codes that reflected key concepts/areas of the CFIR and Kindgon’s multiple streams model of policy change and goals of the parent study. The Principal Investigator (PI) and two qualitative coders then worked collaboratively to refine the coding framework. Initially, the team read three transcripts and identified emergent themes to create a draft codebook of *a priori* and inductive codes. Three transcripts were then coded independently by team members, who then compared the results of their initial coding, refined code definitions, and discussed additional inductive codes. This process was repeated again on three additional transcripts to create the final coding framework, which consisted of 19 codes. Coders then applied the codes systematically to each of the transcripts in MAXQDA software such that each transcript was double coded. Throughout the coding process, the team met weekly to discuss findings, and the PI monitored comparability between coders and resolved discrepancies, ensuring intercoder agreement.

While the parent study focussed broadly on understanding barriers and facilitators to SSP implementation in rural counties in Kentucky, for this analysis, we focussed on examining coded text pertaining to the use of research evidence. We broadly defined research evidence to include any mentions of empirical studies (i.e. research related to SSPs) and governmental reports (i.e. data generated by local, state, or federal agencies). In addition, our research evidence operationalisation encompassed scenarios in which participants described research evidence being ignored or not acted upon by stakeholders. Mentions of research evidence occurred in response to direct questions about the role of research evidence during SSP implementation processes (e.g. “What role did research evidence play in syringe services program implementation?”) and in response to questions that explored SSP implementation processes more broadly (e.g. “Can you walk me through how policy changes occurred to allow syringe services program implementation in your community?”). Research evidence-related quotes were subsequently reviewed and further categorised based on the typologies for applications of research evidence developed by Weiss *et al.* (instrumental, conceptual, and symbolic) as well as a category for instances when research evidence was not used during processes to secure approvals for SSP implementation (i.e. policymakers and other stakeholder groups chose to ignore empirical research evidence) [26,28]. We present our results relative to the typologies developed by Weiss *et al.* given that this analysis aimed to explore applications of research evidence during processes to acquire approvals for SSP implementation. Direct quotes are used in this article to demonstrate how research was applied during processes to acquire approvals for SSP implementation. To protect the anonymity of our study participants, we do not associate quotes with information about where a given participant lives or works, or with detailed descriptions of their specific roles during SSP implementation processes as this information may potentially be identifiable given the rural nature of this study. However, we provide an overview of our participants and their backgrounds in the Results section.

## Results

### Participant characteristics

Eighteen participants completed in-depth interviews (10 women, 8 men), the majority of whom were White (88.9%). Participants reflected a variety of professional roles, including health department and health district directors, healthcare providers, program directors, SSP operators, and persons who provided HIV/AIDS prevention services. In addition, participants described having served their communities in several capacities over their professional and lived experiences, such as *via* involvement with law enforcement, community coalitions, and advisory boards (e.g. at local health departments and non-profits organisations). Several participants reported having been personally affected by the opioid crisis *via* their own histories of drug use or among their friends and families.

### Instrumental research evidence applications

Many participants reported being reticent to support SSPs upon initially learning of their existence; however, research evidence about the public health utility of SSPs dispelled their concerns and ultimately formed the basis for why they pursued program implementation. In these instances, applications of research evidence manifested an instrumental application (i.e. it formed the foundation of why persons chose to support SSP implementation). For example, a participant explained:

*First, I was a little skeptical to be honest. Also, like, ‘Wow, really? We’re going to give needles to drug users?’ I had to become educated, and read, and research, and understand. Once I got that knowledge, received the knowledge, and just really started digging and went through some trainings and webinars and different sessions, I understood the concept.*

Similarly, another participant described their initial reaction to potentially pursuing policy changes in support of SSP implementation:

*I said, ‘I don’t know much about them [SSPs], but I’ll learn about them and then we’ll see.’ So, I did some research. I looked at studies. I went and visited a program… and once you do the research on these programs, they kind of speak for themselves– better health outcomes for the participants, five times more likely to get into treatment, rates of HIV and hepatitis decline.*

Instrumental use of research evidence was also present during processes to acquire approvals for SSP implementation. For example, in some instances, when policymakers learned about the public health benefits of SSP implementation and how vulnerable their communities were to injection drug use-associated infectious disease outbreaks, research evidence was employed instrumentally (i.e. it formed the basis of their decision-making processes) to justify their support for approving SSP implementation. A participant highlighted this instrumental application of research evidence by explaining, “I have to think that it was [Name] County being on the list …on the list of vulnerable counties in the whole Nation. I have to think that that's what tipped the scales. And that it [SSP implementation] has to be this way.” In addition, another participant emphasised that they learned as much as possible about SSPs prior to engaging with policymakers to ensure they were able to effectively communicate research about program implementation and support evidence-based decision making among policymakers:

*You owe it to yourself and to them [policymakers] to research what it is before you offer an opinion. ‘What's the data say? What's the research say? What do we know about this, not just what I think?’ And when you frame it like that, there's not a lot of argument back. I've researched this, I've studied this, I know what I'm talking about…*

### Conceptual research evidence applications

Nearly all participants described having to confront stigmatising and inaccurate beliefs policymakers held about PWID and SSP operations. They described “uphill battles” in efforts to change how stakeholders felt about SSPs, often requiring long periods of sustained outreach and education. In these instances, research evidence was applied conceptually, i.e. participants applied research evidence to change underlying attitudes and beliefs policymakers held that were not evidence-based. For example, a participant explained:

*And so we would have to go to the city councils as well. And that's been an uphill battle because we're having to teach a lot of non-public health folks about syringe services programs and convincing them to drop their stigma and their notions that these programs only enable drug use, and to try to get them to take an honest look at the data that's available, that's been available for decades now, showing that these programs are excellent as far as a public health measure.*

Similarly, another participant stated that their approach to navigating discussions about SSP implementation involved asking stakeholders about their opinions surrounding injection drug use then applying research evidence to correct inaccuracies and change their understanding:

*Most people, I think, come into an idea with preconceived notions of what is happening– biases. And you just, in a calm way, state the facts. Show them what's true, what's not true… You know, ‘You're enabling people who are using drugs by creating these service programs,’ but in truth, it's you're actually helping people get off drugs… So you just kind of lay out the facts to them. That's how I start. I just ask questions to see where they are, why they think that, and then I just lay out the facts.*

### Symbolic research evidence applications

Several participants described employing philosophical approaches to serving vulnerable populations that were aligned with harm reduction prior to learning about the term “harm reduction” and existence of SSPs. Upon learning about the evidence in support of harm reduction and SSPs, some participants applied research evidence in a symbolic fashion to provide legitimacy to the ways in which they already approached public health (e.g. meeting vulnerable persons where they are and supporting the use of public health strategies that fit within the contexts of their life circumstances) and their ultimate support for SSPs. For example, a participant explained:

*I have always been a proponent of taking the service to the people who need that service and who will benefit from it, and recognizing that sometimes it’s not about fixing a person or changing their behavior, but instead doing less harm. So, really, when I started learning, ‘We call that harm reduction,’ okay, I love this, because it’s– so for me, I was very excited and very ready.*

## Non-Applications of research evidence

Many participants described scenarios in which their efforts to acquire approvals for SSP implementation were obstructed by local stakeholders’ unwillingness to consider the implications of research evidence. For example, a participant described an incident in which a member of law enforcement interrupted them during a presentation about risks for injection drug use-associated HIV outbreaks and stated they believed data reported by the Centres for Disease Control and Prevention (CDC) [38] were not factual, “*…I've had a Sheriff actually stand up and say ‘You know that CDC data? It's a lie.’ What do you say with that?”* This participant further explained that a subsequent slide in their presentation detailed the risk vulnerability analyses and that the law enforcement official was unwilling to consider it as factual:

*…fortunately, my next slide was to show them what their county rankings were on those six variables that the CDC used in the vulnerability study and try to get them to recognize that ‘Your county looks like Scott County, Indiana, based on all of these variables.’ That didn't convince him, though. I mean, he had made his mind up. This [SSP implementation] wasn't going to happen in his community.*

Another participant shared the sentiment that many persons were simply unwilling to consider empirical research evidence in support of SSP implementation:

*So, you know, you've got some people out there that you're not going to convince [about the need for SSPs]. Realize, you know, you just have to let them voice this and you're not going to educate them about it because they've got their feeling and they don't want to be confused with the facts. They've made up their mind and that's where they're at.*

## Discussion

Applications of research evidence during processes to acquire approvals for SSP implementation in rural counties in Kentucky were heterogeneous in nature. For example, instrumental applications of research evidence occurred at the intrapersonal level to allay participants’ initial concerns about SSP implementation and form the basis for why they ultimately pursued program implementation. At the interpersonal level, instrumental use of research evidence also occurred when proponents for SSP implementation educated policymakers about the need for SSPs who subsequently used the information they gleaned as the basis for their decisions to support program approval. In addition, proponents for SSP implementation applied research evidence in a conceptual fashion to address underlying attitudes and beliefs policymakers held that were not grounded in facts. Participants also reported symbolic applications of research evidence to justify their pre-existing attitudes and beliefs about meeting the public health needs of PWID in ways that aligned with harm reduction- and SSP-related research. Similar to existing research [26], we also found instances in which lay stakeholders viewed research evidence with scepticism and an unwillingness to consider its merits. This research builds on existing drug policy literature by demonstrating the importance of understanding the ways in which research evidence can be applied to support the implementation of evidence-based interventions that may be misunderstood or stigmatised by lay stakeholders, especially in a rural context.

Participants routinely described how the results of a widely disseminated study that identified 220 counties vulnerable to injection drug use-associated infectious disease outbreaks served as a cue to action for SSP implementation [38]. For example, in some instances, when policymakers learned of the vulnerability of their counties, it motivated them to act on the evidence in an instrumental fashion and support SSP implementation. Given that the vulnerability analyses were published in 2015 and that more than 70 SSPs have been implemented across Kentucky in the past six years [38,43], this finding demonstrates the power of applying research evidence to emerging public health threats as well as the importance of ensuring empirical data resonate with lay stakeholders and are easily understood. Future work should be conducted to better understand how to tailor messaging about scientific findings to diverse audiences and ensure persons are able to learn about specific evidence-based response strategies that may provide public health benefits.

Among participants, many described applying research evidence conceptually to change how local stakeholders perceived SSPs. Notably, interviewees elaborated that changing the hearts and minds of persons who were initially against SSP implementation can be a lengthy process that involves information generated from research, but also confronting biases and stigma. While these successes should not be discounted, future work is needed to better understand how to expedite processes to educate policymakers about emerging public health threats and evidence-based response strategies. Given rapid escalations in injection drug use-associated morbidity and mortality [44], shortening the amount of time required for policymakers to act on research evidence in support of SSP implementation may result in significant public health gains.

Proponents for SSP implementation described symbolic applications of research evidence in that, upon learning about harm reduction- and SSP-related research, they were afforded legitimisation of their pre-existing ideas and approaches to public health (i.e. working to meet persons where they are and encouraging them to utilise public health strategies that fit within their lives). This application of research evidence is notable by itself, but also because it demonstrates that many participants were unaware of empirical studies that supported their approaches to public health. While this instance of symbolic use of research evidence did not directly apply to policy change processes, it likely empowered SSP proponents to engage local stakeholders in discussions about SSP implementation and subsequently apply research evidence in other manners (e.g. instrumentally, conceptually). Future work should more closely examine the constellation of factors (e.g. motivators, cues to action, applications of research evidence) that must align among the public health workforce to propel the implementation of evidence-based response strategies.

Similar to a 2015 study conducted in three urban areas [26], participants described that processes to acquire approvals for SSP implementation were obstructed by policymakers who were unwilling to consider the merits of research evidence. This reluctance to act on research evidence varied considerably; for example, some participants described interacting with stakeholders who had personal beliefs (often driven by inaccurate fears about SSPs and stigmatisation of drug use) that motivated their opposition to SSPs and inaction on empirical evidence. In other scenarios, participants described interacting with community members who voiced doubts about the scientific merits of program implementation. Additional research is warranted to explore strategies to ensure empirical evidence is at the foundation of public health decision-making processes and build trust in scientific findings among diverse stakeholder groups.

There is widespread consensus among the scientific community that SSPs carry substantial public health benefits, yet the translation of research findings to the policy and practice realms remains challenging, even during a national surge of overdose fatalities and increasing risks for injection drug use associated-outbreaks of bloodborne infections [7,38,44]. Our findings build on existing literature by documenting that while rural counties in Kentucky have had great success in rapidly increasing the number of operational SSPs, proponents have faced many barriers to program implementation. Additional study is warranted to explore how diverse stakeholder groups can work together during crises to achieve evidence-based solutions.

This research has several strengths and limitations that should be considered. Our first strength is that we interviewed persons with diverse roles during SSP implementation processes in rural counties across Kentucky, enhancing how we understood applications of research evidence. Another strength is that the proliferation of SSPs in Kentucky was a relatively recent phenomena, potentially limiting recall bias. Among the limitations of our study, our participants reflected the perspectives of persons who were ultimately supportive of SSP implementation. As such, we did not hear firsthand accounts of persons who remain in opposition to program operations. That said, many of our participants described being reluctant to support SSPs upon first learning about them and the evolution of their perspective. Our study results should also be interpreted with consideration for relevant state policy context. The state-level legislation that allowed for SSP implementation, for example, required program operators to secure three levels of approval prior to launching a program. This requirement may limit the generalisability of our results to other contexts in which approvals for program operations are not required. Relatedly, our findings are only reflective of rural communities in Kentucky; similar studies should be conducted in rural communities throughout the US given that the manifestations of research evidence during SSP implementation processes may be heterogeneous in nature. For example, the degree to which stakeholders perceive public health agencies and organisations as credible, and by extension the research evidence they produce, may vary. Additionally, exploring the role of misinformation during SSP implementation processes may yield important insights that can inform program scale up. Finally, all participants were offered an incentive to participate in this research. As a result, our findings may contain some level of bias; however, this potential limitation is likely minor given that our incentive was relatively small in value and all of our participants reflected persons who were known to play a role in SSP implementation. These limitations notwithstanding, this study offers insight into the ways research evidence can be used during processes to implement SSPs.

Public health decisions should be made based on empirical research evidence; however, applications of research evidence during processes to secure approvals for SSP implementation manifested differently in rural counties in Kentucky. In some instances, research evidence formed the basis for expeditious decision-making processes related to SSP implementation, but in other cases, the application of research evidence was slow and involved changing how persons perceived SSPs and overcoming pre-existing stigmatisation of substance use. Expediting the translation of research evidence to the policy realm and supporting efforts to bring SSPs to scale is of the utmost importance given the magnitude of the opioid overdose crisis.

## Data Availability

The data used in this research are not publicly available due to concerns about confidentiality; however, we summarise descriptive information about our participants in the manuscript text. The data that support the findings of this study are available from Dr. Sean T. Allen upon reasonable request.

## References

[1] Larney S, Leung J, Grebely J, et al. Global systematic review and ecological analysis of HIV in people who inject drugs: National population sizes and factors associated with HIV prevalence. Int J Drug Policy. 2020;77:102656. 31951926 10.1016/j.drugpo.2019.102656

[2] Degenhardt L, Peacock A, Colledge S, et al. Global prevalence of injecting drug use and sociodemographic characteristics and prevalence of HIV, HBV, and HCV in people who inject drugs: a multistage systematic review. Lancet Glob Health. 2017;5(12) :e1192–e1207. 29074409 10.1016/S2214-109X(17)30375-3 PMC5683738

[3] Rashti R, Alavian SM, Moradi Y, et al. Global prevalence of HCV and/or HBV coinfections among people who inject drugs and female sex workers who live with HIV/AIDS: a systematic review and Meta-analysis. Arch Virol. 2020;165(9) :1947–1958. 32617764 10.1007/s00705-020-04716-1

[4] Leung J, Peacock A, Colledge S, et al. A global Meta-analysis of the prevalence of HIV, hepatitis C virus, and hepatitis B virus among people who inject Drugs-Do Gender-Based differences vary by Country-Level indicators? J Infect Dis. 2019;220(1) :78–90. 30726973 10.1093/infdis/jiz058 PMC6775227

[5] Broz D, Wejnert C, Pham HT, et al.; National HIV Behavioral Surveillance System Study Group. HIV infection and risk, prevention, and testing behaviors among injecting drug users – national HIV behavioral surveillance system, 20 U.S. cities, 2009. MMWR Surveill Summ. 2014;63(6):1–51. 24990587

[6] Hagan H, Pouget ER, Williams IT, et al. Attribution of hepatitis C virus seroconversion risk in young injection drug users in 5 US cities. J Infect Dis. 2010;201(3):378–385. 20053137 10.1086/649783

[7] CDC. Syringe Services Programs (SSPs). 2019 5-23-19 [cited 2020 9-21-20]. https://www.cdc.gov/ssp/index.html.

[8] Des Jarlais DC, Nugent A, Solberg A, et al. Syringe service programs for persons who inject drugs in urban, suburban, and rural Areas - United States, 2013. MMWR Morb Mortal Wkly Rep. 2015;64(48):1337–1341. 26655918 10.15585/mmwr.mm6448a3

[9] Ruiz MS, O'Rourke A, Allen ST. Impact evaluation of a policy intervention for HIV prevention in Washington, DC. AIDS Behav. 2016;20(1) :22–28. 26336945 10.1007/s10461-015-1143-6 PMC4715855

[10] Ruiz MS, Oʼrourke A, Allen ST, et al. Using interrupted time series analysis to measure the impact of legalized syringe exchange on HIV diagnoses in baltimore and philadelphia. J Acquir Immune Defic Syndr. 2019;82(Suppl 2):S148–s154. 31658203 10.1097/QAI.0000000000002176 PMC6820712

[11] Aspinall EJ, Nambiar D, Goldberg DJ, et al. Are needle and syringe programmes associated with a reduction in HIV transmission among people who inject drugs: a systematic review and Meta-analysis. Int J Epidemiol. 2014;43(1) :235–248. 24374889 10.1093/ije/dyt243

[12] Fernandes RM, Cary M, Duarte G, et al. Effectiveness of needle and syringe programmes in people who inject drugs - An overview of systematic reviews. BMC Public Health. 2017;17(1):309. 28399843 10.1186/s12889-017-4210-2 PMC5387338

[13] Hagan H, McGough JP, Thiede H, et al. Reduced injection frequency and increased entry and retention in drug treatment associated with needle-exchange participation in seattle drug injectors. J Subst Abuse Treat. 2000;19(3):247–252. 11027894 10.1016/s0740-5472(00)00104-5

[14] Platt L, Minozzi S, Reed J, et al. Needle syringe programmes and opioid substitution therapy for preventing hepatitis C transmission in people who inject drugs. Cochrane Database Syst Rev. 2017;9(9):Cd012021. 28922449 10.1002/14651858.CD012021.pub2 PMC5621373

[15] Bornstein KJ, Coye AE, St Onge JE, et al. Hospital admissions among people who inject opioids following syringe services program implementation. Harm Reduct J. 2020;17(1):30. 32398059 10.1186/s12954-020-00376-1 PMC7216361

[16] Des Jarlais DC. Harm reduction in the USA: the research perspective and an archive to david purchase. Harm Reduct J. 2017;14(1):51. 28747189 10.1186/s12954-017-0178-6 PMC5530540

[17] Allen ST, Grieb SM, O'Rourke A, et al. Understanding the public health consequences of suspending a rural syringe services program: a qualitative study of the experiences of people who inject drugs. Harm Reduct J. 2019;16(1):33. 31109339 10.1186/s12954-019-0305-7 PMC6528286

[18] Sherman SG, Purchase D. Point defiance: a case study of the United States' first public needle exchange in tacoma, Washington. Int J Drug Policy. 2001;12(1):45–57. 11275503 10.1016/s0955-3959(00)00074-8

[19] Allen ST, Ruiz MS, Jones J. Quantifying syringe exchange program operational space in the District Of Columbia. AIDS Behav. 2016;20(12):2933–2940. 27094786 10.1007/s10461-016-1405-y PMC5108823

[20] Allen ST, Ruiz MS, Jones J, et al. Legal space for syringe exchange programs in hot spots of injection drug use-related crime. Harm Reduct J. 2016;13:16. 27112328 10.1186/s12954-016-0104-3 PMC4845437

[21] Jones CM. Syringe services programs: an examination of legal, policy, and funding barriers in the midst of the evolving opioid crisis in the U.S. Int J Drug Policy. 2019;70:22–32. 31059965 10.1016/j.drugpo.2019.04.006

[22] Weinmeyer R. Needle exchange programs' status in US politics. AMA J Ethics. 2016;18(3) :252–257. 27002996 10.1001/journalofethics.2016.18.3.hlaw1-1603

[23] Rich JD, Adashi EY. Ideological anachronism involving needle and syringe exchange programs: Lessons from the Indiana HIV outbreak. Jama. 2015;314(1):23–24. 26000661 10.1001/jama.2015.6303 PMC4496270

[24] Fernández-Viña MH, Prood NE, Herpolsheimer A, et al. State laws governing syringe services programs and participant syringe possession, 2014-2019. Public Health Rep. 2020;135(1_suppl):128S–137s. 32735195 10.1177/0033354920921817 PMC7407055

[25] Anderson W. The New York needle trial: the politics of public health in the age of AIDS. Am J Public Health. 1991;81(11):1506–1517. 1951815 10.2105/ajph.81.11.1506 PMC1405677

[26] Allen ST, Ruiz MS, O'Rourke A. The evidence does not speak for itself: the role of research evidence in shaping policy change for the implementation of publicly funded syringe exchange programs in three US cities. Int J Drug Policy. 2015;26(7):688–695. 25979789 10.1016/j.drugpo.2015.04.008 PMC4468034

[27] Weiss CH, Murphy-Graham E, Birkeland S. An alternate route to policy influence:How evaluations affect D.A.R.E. American Journal of Evaluation. 2005;26(1):12–30.

[28] Weiss CH. The many meanings of research utilization. Public Administration Review. 1979;39(5):426–431.

[29] Amara N, Ouimet M, Landry R. New evidence on instrumental, conceptual, and symbolic utilization of university research in government agencies. Science Communication. 2004;26(1):75–106.

[30] Cousins JB, Leithwood KA. Current empirical research on evaluation utilization. Review of Educational Research. 1986;56(3):331–364.

[31] Weiss JA, Weiss CH. Social scientists and decision makers look at the usefulness of mental health research. Am Psychol. 1981;36(8):837–847. 7294491 10.1037//0003-066x.36.8.837

[32] Leviton LC, Hughes EFX. Research on the utilization of evaluations:a review and synthesis. Eval Rev. 1981;5(4):525–548.

[33] Keyes KM, Cerdá M, Brady JE, et al. Understanding the rural-urban differences in nonmedical prescription opioid use and abuse in the United States. Am J Public Health. 2014;104(2):e52–e59. 24328642 10.2105/AJPH.2013.301709 PMC3935688

[34] Monnat SM, Rigg KK. Examining Rural/Urban Differences in Prescription Opioid Misuse Among US Adolescents . J Rural Health. 2016;32(2):204–218. 26344571 10.1111/jrh.12141 PMC4779738

[35] Rigg KK, Monnat SM, Chavez MN. Opioid-related mortality in rural america: Geographic heterogeneity and intervention strategies. Int J Drug Policy. 2018;57:119–129. 29754032 10.1016/j.drugpo.2018.04.011

[36] Rigg KK, Monnat SM. Urban vs. rural differences in prescription opioid misuse among adults in the United States: informing region specific drug policies and interventions. Int J Drug Policy. 2015;26(5):484–491. 25458403 10.1016/j.drugpo.2014.10.001 PMC4397122

[37] Weiss AJ, et al. Opioid-Related inpatient stays and emergency department visits by state, 2009–2014: Statistical brief #219, in healthcare cost and utilization project (HCUP) statistical briefs. Rockville (MD): Agency for Healthcare Research and Quality (US); 2006. 28682575

[38] Van Handel MM, Rose CE, Hallisey EJ, et al. County-Level vulnerability assessment for rapid dissemination of HIV or HCV infections among persons who inject drugs, United States. J Acquir Immune Defic Syndr. 2016;73(3):323–331. 27763996 10.1097/QAI.0000000000001098 PMC5479631

[39] Kentucky Cabinet for Health and Family Services. Syringe Exchange Programs. 7-20-21]; 2017. https://chfs.ky.gov/agencies/dph/dehp/hab/Pages/kyseps.aspx

[40] Morse JM. The significance of saturation. Qual Health Res. 1995;5(2):147–149.

[41] CFIR Research Team-Center for Clinical Management Research. Consolidated Framework for Implementation Research. 7-20-21]; 2022. https://cfirguide.org/

[42] Kingdon JW. Agendas, alternatives, and public policies. Boston: Little, Brown; 1984.

[43] Kentucky Cabinet for Health and Family Services. Syringe Exchange Programs; 2017.

[44] CDC. 2018 Drug Overdose Death Rates. 2020.

